# Community-Acquired Solitary Brain Abscesses Caused by Hypervirulent *Klebsiella pneumoniae* in a Healthy Adult

**DOI:** 10.3390/microorganisms12050894

**Published:** 2024-04-29

**Authors:** Joo-Hee Hwang, Jung Soo Park, Tae Won Bae, Jeong-Hwan Hwang, Jaehyeon Lee

**Affiliations:** 1 Department of Internal Medicine, Jeonbuk National University Medical School and Hospital, Jeonju 54907, Republic of Korea; j.hwang@jbnu.ac.kr; 2 Research Institute of Clinical Medicine of Jeonbuk National University—Biomedical Research Institute of Jeonbuk National University Hospital, Jeonju 54907, Republic of Korea; 3 Department of Neurosurgery, Jeonbuk National University Medical School and Hospital, Jeonju 54907, Republic of Korea; rollingstone12@hanmail.net; 4 Department of Laboratory Medicine, Presbyterian Medical Center, Jeonju 54987, Republic of Korea; baetw@jbnu.ac.kr; 5 Department of Laboratory Medicine, Jeonbuk National University Medical School and Hospital, Jeonju 54907, Republic of Korea

**Keywords:** hypervirulent *Klebsiella pneumoniae*, community-acquired infection, brain abscess, virulence

## Abstract

A 42-year-old man was admitted to the emergency room complaining of fever and headache. His cerebrospinal fluid showed a cloudy appearance, and his white blood cell count was elevated at 2460/mm^3^, with a predominance of neutrophils (81%), and abnormal protein and glucose levels (510.7 mg/dL and 5 mg/dL, respectively). A lobulated lesion with rim enhancement, suggestive of abscess, was detected through magnetic resonance imaging. *Klebsiella pneumoniae* was detected in nasopharyngeal swab and blood cultures. The capsular serotype of *K. pneumoniae* was K2 and the sequence type determined by multilocus sequence typing was 23. The hypervirulent phenotype was associated with multiple virulent genes, including *rmpA*, *rmpA2*, *entB*, *ybtS*, *kfu*, *iucA*, *iutA*, *iroB mrkD*, *allS*, *peg-344*, *peg-589*, and *peg-1631*. After six weeks of receiving appropriate antibiotics and exhibiting clinical resolution of the brain abscesses, the patient was discharged. We present the first reported case of a healthy community-dwelling adult with solitary brain abscesses, and no other invasive abscesses, related to hypervirulent *K. pneumoniae*.

## 1. Introduction

Brain abscess is a life-threatening intracerebral infection that can arise from contiguous infections such as otitis or sinusitis, neurosurgery, traumatic brain injury, or hematogenous spread [1]. While *Streptococcus* and *Staphylococcus* spp. are the most common pathogens involved in brain abscesses, *Klebsiella pneumoniae* is an extremely rare cause of brain abscess, implicated in only 1–2% of cases in Western countries [2]. *K. pneumoniae* is, however, more frequently identified in Asia, where it has been found in about 10% of cases and reported as an increasingly important cause of brain abscess in Taiwan [2]. There are currently two types of *K. pneumoniae* in circulation: “classic” *K. pneumoniae* (cKp) and hypervirulent *K. pneumoniae* (hvKp). Clinically, cKp is known to cause opportunistic infections primarily in immunocompromised patients in healthcare settings. However, hvKp is widely considered a more virulent pathogen in community settings [3]. HvKp infections are characterized by their ability to affect individuals of any age with relatively good health, and their propensity to cause multiple infections at various anatomical sites [3].

Although the characteristics of hvKp and its differences from cKp are relatively unknown, at least 78 capsular serotypes, including K1, K2, K5, K16, K20, K54, K57, and KN1, are the most common highly pathogenic serotypes [4]. A recent report has demonstrated that serotypes K1 and K2 are predominantly linked with hvKp [3]. The specific definition of hvKp remains uncertain; nevertheless, Russo et al. have evidenced that *iroB*, *iucA*, *peg-344*, *rmpA*, and *rmpA2* serve as markedly disparate virulence determinants, endowing a hypervirulent phenotype [5]. Currently, *iuc*, *rmpA*, and *rmpA2* stand out as the most thoroughly investigated molecular markers of hvKp [6].

Despite the increasing prevalence of hvKp infection in Taiwan, especially since the mid-1980s, cases of community-acquired solitary brain abscess attributed to *K. pneumoniae* are rare, leaving our understanding of the association between brain abscess and *K. pneumoniae* limited [4,7]. Moreover, the clinical significance of hvKp-related K1 or K2 capsular serotypes or virulence genes in brain abscesses remains unclear. Here, we present for the first time the case of a healthy community-dwelling adult diagnosed with brain abscesses associated with hvKp, exhibiting identified virulence factors and capsule type, and without other invasive abscesses.

## 2. Materials and Methods

### 2.1. Bacterial Identification and Antimicrobial Susceptibility Test

All laboratory tests were performed in routine clinical practice. Blood cultures were taken with Bact/Alert Culture Media (FA Plus, FN Plus, BacT/Alert 3D system, bioMérieux, Durham, NC, USA). Isolates from blood culture bottles were inoculated to blood agar plates (POAMEDIA Blood Agar Plates, Shin Yang Chemical Co., Seoul, Republic of Korea) and MacConkey agar plates (POAMEDIA MacConkey Agar Plates, Shin Yang Chemical Co., Seoul, Republic of Korea) in a 5% CO_2_ incubator for 16 to 24 h at 35 °C. Microorganisms were identified using the Vitek MS system (bioMérieux, Hazelwood, MI, USA), and antimicrobial susceptibility tests were performed using VITEK 2 identification systems with AST 211 cards (bioMérieux, Marcy-l’Étoile, France) [8]. Antimicrobials used for the test included aminoglycosides (amikacin, gentamicin), cephalosporins (cefazolin, cefotaxime, ceftazidime, cefepime), cephamycins (cefoxitin), fluoroquinolones (levofloxacin), folate pathway inhibitors (trimethoprim-sulfamethoxazole), glycylcyclines (tigecycline), monobactams (aztreonam), and penicillin + β lactamase inhibitors (ampicillin–sulbactam, piperacillin–tazobactam).

### 2.2. PCR-Based Detection of Virulence Genes and Multilocus Sequence Typing (MLST)

All the molecular tests were conducted in the same way as previously reported [9]. In short, DNA was extracted from *K. pneumoniae* isolate using the boiling method and extraction buffer (Seegene, Seoul, Republic of Korea) [10]. Two to three colonies from a blood agar plate were transferred to 1 mL of distilled water in an Eppendorf tube. The mixture was then vortexed and centrifuged at 13,000 rpm for 10 min using a microcentrifuge. The supernatant was then carefully removed and 100 μL of DNA extraction solution was introduced into the pellet. After another round of vortexing, the sample was heated at 95 °C for 20 min. After incubation, it was centrifuged once again at 13,000 rpm for 10 min in a microcentrifuge. The supernatant was used for PCR. K1 and K2 capsular serotypes, and seven virulence genes (*rmpA*, *entB*, *ybtS*, *kfu*, *iutA*, *mrkD*, and *allS*) were detected by multiplex PCR using the Qiagen multiplex PCR kit (Qiagen, Courtaboeuf, France). Primer sets for *magA* (*wzy*-like polymerase specific to K1 strain), K2 capsular serotype-specifying *wzi* gene, and other virulence genes (*rmpA*, *entB*, *ybtS*, *kfu*, *iutA*, *mrkD*, and *allS*) were used as previously described [11]. Other serotypes, including K5, K20, K54, and K57, and other virulence genes, including *rmpA*, *rmpA2*, *iucA*, *iroB*, *peg-344*, *peg-589*, and *peg-1631*, were analyzed using EmeraldAmp PCR Master Mix (Takara Bio Inc., Shiga, Japan) as previously described [5,12]. The isolate was sent to Macrogen (Seoul, Republic of Korea) for MLST, and sequencing and analysis were performed for seven housekeeping genes (*gapA*, *infB*, *mdh*, *pgi*, *phoE*, *rpoB*, and *tonB*) [13].

## 3. Case Presentation

A 42-year-old male presented to the emergency department complaining of fever and headache, accompanied by nausea and vomiting, for one week. His initial blood pressure was 136/96 mmHg, pulse rate was 78 beats/min, respiratory rate was 20 breaths/min, percutaneous oxygen saturation was 99% with room air, and body temperature was 38.7 °C. Upon neurological examination, the patient was confused, with a Glasgow Coma Scale score of 13 (E3V4M6). He had neck stiffness with no other evidence of meningeal irritation. He had normal limb muscular strength and normal deep and superficial sensations.

**Figure 1:**
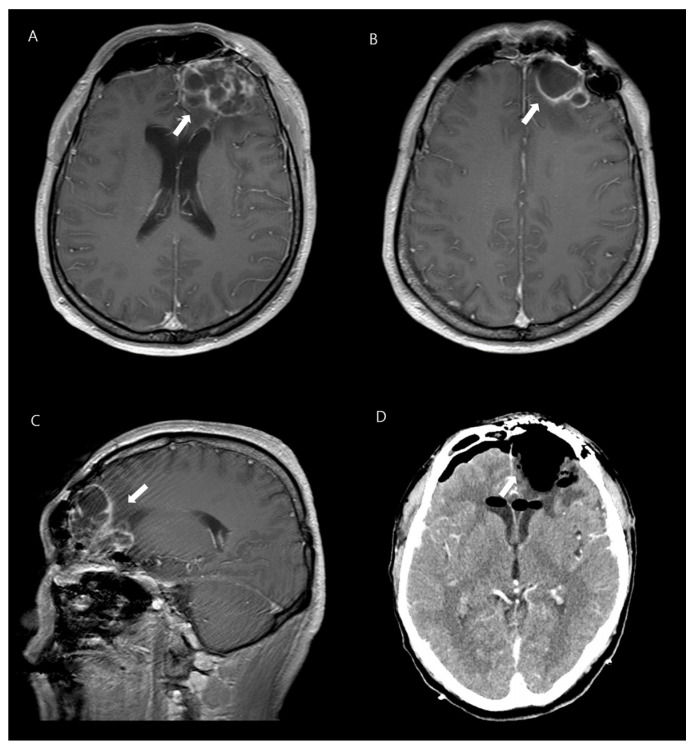
A 42-year-old man with *K. pneumoniae* brain abscesses. Axial contrast-enhanced T1-weighted images (**A**,**B**) and sagittal image reveal thin-walled rim-enhancing abscesses in the left frontal lobe (**C**). Enhanced brain CT shows disappeared lesions after the neurosurgery (**D**).

Further investigations, including a lumbar puncture, were performed. The patient’s biochemistry and hematology panel showed normal liver and renal function, as well as an absence of leukocytosis with a normal differential cell count, except for elevated levels of high-sensitivity C-reactive protein (109.4 mg/L) and procalcitonin (1.98 ng/mL). Urinalysis was normal. Analysis of cerebrospinal fluid (CSF) obtained through lumbar puncture showed a cloudy appearance; elevated white blood cell count of 2460/mm^3^, with a predominance of neutrophils (81%); and abnormal protein and glucose levels (510.7 mg/dL and 5 mg/dL, respectively). The patient’s intracranial pressure was found to be elevated at 190 mmH_2_O. Gram staining of the CSF did not show any microorganisms and cultures were negative. However, blood cultures showed *K. pneumoniae* growth. Nasopharyngeal swab culture was taken to identify *K. pneumoniae* colonization, and it tested positive. A chest X-ray; computed tomography (CT) scan of the chest, abdomen, and pelvis; electrocardiogram; and transthoracic echocardiogram were performed, all of which revealed no abnormalities. However, magnetic resonance imaging (MRI) of his brain revealed a lobulated lesion with rim enhancement, suggestive of abscess, in the left frontal area and craniotomy site, and showed evidence of ventriculitis and empyema in both lateral ventricles (Figure 1).

The patient had no significant medical history, was not taking any prescription medications, and did not have any risk factors for meningitis, including diabetes mellitus, underlying malignancy, leukemia, human immunodeficiency virus, autoimmune disease, organ transplants, or long-term steroid use. Twenty-six years prior to the current admission, he had a craniotomy due to a traumatic intracranial hemorrhage in a car crash. Upon examination, the patient had no evidence of otitis media, sinusitis, pharyngitis, or mastoiditis. Furthermore, he had not received any dental treatments for in the past year and had no evidence of tooth decay or tooth extraction. 

He was started on empiric treatment for brain abscess with intravenous vancomycin, ceftriaxone, and metronidazole. The neurosurgery team performed a craniotomy to remove the abscess and inserted an external ventricular drain. Culture of the abscess pus yielded *K. pneumoniae*. The PCR results for capsular serotype showed K2, and the MLST result was ST23. The PCR test also detected presence of all thirteen virulence genes (*rmpA*, *rmpA2*, *entB*, *ybtS*, *kfu*, *iucA*, *iutA*, *iroB*, *mrkD*, *allS*, *peg-344*, *peg-589*, and *peg-1631*). Following the initial empiric treatment, de-escalation to ceftriaxone was ordered based on conclusive susceptibilities. After six weeks of parenteral antibiotics, the patient showed signs of clinical resolution of the brain abscess and was discharged home.

## 4. Discussion

We report a case of solitary brain abscesses caused by hvKp in a previously healthy community-dwelling adult with no known risk factors for infection. This case highlights an important clinical finding: hvKp can cause primary brain abscess in otherwise healthy adults. To our knowledge, this is the first study to investigate capsular serotypes and virulence factors in *K. pneumoniae*-related brain abscesses.

*K. pneumoniae* can colonize various mucosal surfaces in humans, including the gastrointestinal tract and nasopharynx, following acquisition [14]. The prevalence of *K. pneumoniae* intestinal colonization among healthy individuals from the community in Western countries ranges from 5% to 23% [15,16,17]. In Asia, high rates of gut colonization have been observed, ranging from 19% to 88% [18]. The rates of community-acquired colonization of the nasopharynx have been reported to range from 3 to 15% [19,20,21]. 

Since a rectal culture was not performed, gut colonization could not be confirmed in this patient. However, there was evidence of *K. pneumoniae* colonization in the nasopharynx. In addition, *K. pneumoniae* was isolated in the peripheral blood and the pus specimens obtained from neurosurgery. The chest and abdominopelvic CT did not show any foci of infection. Additionally, the MRI gave no indications of head and neck infections. Given the limited evidence, the potential for a contagious route of *K. pneumoniae* in this patient is being considered. Since the patient has *K. pneumoniae* colonization, it is likely that the infection originated in the nasopharynx. We presume that hvKp formed colonies in the nasopharynx and migrated to the brain through a broken anatomical barrier. The migrated hvKp contributed to the formation of the brain abscess and then became systemic.

Brain abscesses caused by Gram-negative organisms are often associated with nosocomial infections, particularly in patients with impaired immune function. It was determined that he did not have a postoperative nosocomial infection because he had been in the community for a long time, even though there was a broken anatomical barrier remaining. Although the patient did not have DM or alcoholism, we ultimately conclude that the continued presence of a broken anatomical barrier constituted a risk factor for *K. pneumoniae* brain abscess.

The endemicity of hvKp is mainly restricted to Asian countries, and the prevalent STs by MLST are ST23, ST26, ST57, ST65, ST86, ST163, and ST375 [22]. ST23 is the most prevalent among hvKp isolates, with a prevalence from 30 to 85% [22]. Our hvKp isolate also belongs to ST23. Furthermore, in this instance, the capsular serotype identified was K2. In a previous study, the majority of K1 isolates were associated with ST23, whereas K2 isolates were distributed across more than 10 different ST types, with ST65/ST86 being the predominant type [23]. However, in our recent study of *K. pneumoniae*-related renal abscesses, we observed that ST23 was primarily associated with K1, but also with K2 [9]. The hypervirulent phenotype exhibited by our isolate was associated with multiple virulence genes, including *rmpA*, *rmpA2*, *entB*, *ybtS*, *kfu*, *iucA*, *iutA*, *iroB*, *mrkD*, *allS*, *peg-344*, *peg-589*, and *peg-1631*. Until recently, a clear definition of hvKp has been controversial. Hypervirulence is not regarded as arising from a single gene, but rather is regarded as the result of a series of complex interactions between multiple virulence determinants [24]. To date, several virulence factors, including *iuc*, *rmpA*, and *rmpA2*, have emerged as the most comprehensively understood virulence factors based on previous studies [6].

Invasive community-acquired infections can arise from hvKp even in immunocompetent adults and often involve multiple anatomical sites [4]. In this study, the case of solitary brain abscesses caused by hvKp was community-acquired. Bacteremia, multiorgan abscess formation, and metastatic spread are well-known features of hvKp [4,25,26]. Although bacteremia was evident in our case of hvKp-related brain abscesses, multiorgan involvement was not observed. Nonetheless, considering prior data, it is advisable to evaluate for the presence of multiorgan abscess formation in patients with brain abscess and bacteremia caused by hvKp.

## 5. Conclusions

We report the case of hvKp-related solitary brain abscesses in an immunocompetent patient, acquired from the community and in the absence of other metastatic septic abscesses. While *K. pneumoniae* is known as an important hospital-acquired pathogen that poses a risk to immunocompromised patients who develop brain abscess, clinicians should now consider hvKp as a causative pathogen in community-acquired brain abscess in immunocompetent patients.

## Data Availability

The data presented in this study are available on request from the corresponding author. The data are not publicly available due to privacy restrictions.

## References

[1] Cantiera M., Tattevin P., Sonneville R. (2019). Brain abscess in immunocompetent adult patients. Rev. Neurol..

[2] Brouwer M.C., Coutinho J.M., van de Beek D. (2014). Clinical characteristics and outcome of brain abscess: Systematic review and meta-analysis. Neurology.

[3] Paczosa M.K., Mecsas J. (2016). Klebsiella pneumoniae: Going on the offense with a strong defense. Microbiol. Mol. Biol. Rev..

[4] Shon A.S., Bajwa R.P., Russo T.A. (2013). Hypervirulent (hypermucoviscous) Klebsiella pneumoniae: A new and dangerous breed. Virulence.

[5] Liu C., Guo J. (2018). Characteristics of ventilator-associated pneumonia due to hypervirulent Klebsiella pneumoniae genotype in genetic background for the elderly in two tertiary hospitals in China. Antimicrob. Resist. Infect. Control.

[6] Russo T.A., Marr C.M. (2019). Hypervirulent Klebsiella pneumoniae. Clin. Microbiol. Rev..

[7] Zhao J., Huo T., Luo X., Lu F., Hui S., Yang B. (2022). Klebsiella pneumoniae-related brain abscess and meningitis in adults: Case report. Medicine.

[8] (2018). Performance Standards for Antimicrobial Susceptibility Testing.

[9] Lee J., Hwang J.-H., Yeom J.H., Lee S., Hwang J.-H. (2024). Analysis of virulence profiles in clinical isolates of Klebsiella pneumoniae from renal abscesses: Clinical significance of hypervirulent isolates. Front. Cell. Infect. Microbiol..

[10] Ivanov I.G., Bachvarov D.R. (1987). Determination of plasmid copy number by the “boiling” method. Anal. Biochem..

[11] Compain F., Babosan A., Brisse S., Genel N., Audo J., Ailloud F., Kassis-Chikhani N., Arlet G., Decré D. (2014). Multiplex PCR for detection of seven virulence factors and K1/K2 capsular serotypes of Klebsiella pneumoniae. J. Clin. Microbiol..

[12] Russo T.A., Olson R., Fang C.-T., Stoesser N., Miller M., MacDonald U., Hutson A., Barker J.H., La Hoz R.M., Johnson J.R. (2018). Identification of biomarkers for differentiation of hypervirulent Klebsiella pneumoniae from classical K. pneumoniae. J. Clin. Microbiol..

[13] Diancourt L., Passet V., Verhoef J., Grimont P.A., Brisse S. (2005). Multilocus sequence typing of Klebsiella pneumoniae nosocomial isolates. J. Clin. Microbiol..

[14] Podschun R., Ullmann U. (1998). Klebsiella spp. as nosocomial pathogens: Epidemiology, taxonomy, typing methods, and pathogenicity factors. Clin. Microbiol. Rev..

[15] Gorrie C.L., Mirčeta M., Wick R.R., Edwards D.J., Thomson N.R., Strugnell R.A., Pratt N.F., Garlick J.S., Watson K.M., Pilcher D.V. (2017). Gastrointestinal carriage is a major reservoir of Klebsiella pneumoniae infection in intensive care patients. Clin. Infect. Dis..

[16] Martin R.M., Cao J., Brisse S., Passet V., Wu W., Zhao L., Malani P.N., Rao K., Bachman M.A. (2016). Molecular epidemiology of colonizing and infecting isolates of Klebsiella pneumoniae. MSphere.

[17] Thom B. (1970). Klebsiella in faeces. Lancet.

[18] Lin Y.-T., Siu L.K., Lin J.-C., Chen T.-L., Tseng C.-P., Yeh K.-M., Chang F.-Y., Fung C.-P. (2012). Seroepidemiology of Klebsiella pneumoniae colonizing the intestinal tract of healthy Chinese and overseas Chinese adults in Asian countries. BMC Microbiol..

[19] Dao T.T., Liebenthal D., Tran T.K., Ngoc Thi Vu B., Ngoc Thi Nguyen D., Thi Tran H.K., Thi Nguyen C.K., Thi Vu H.L., Fox A., Horby P. (2014). Klebsiella pneumoniae oropharyngeal carriage in rural and urban Vietnam and the effect of alcohol consumption. PLoS ONE.

[20] Farida H., Severin J.A., Gasem M.H., Keuter M., van den Broek P., Hermans P.W., Wahyono H., Verbrugh H.A. (2013). Nasopharyngeal carriage of Klebsiella pneumoniae and other Gram-negative bacilli in pneumonia-prone age groups in Semarang, Indonesia. J. Clin. Microbiol..

[21] Davis T.J., Matsen J.M. (1974). Prevalence and characteristics of Klebsiella species: Relation to association with a hospital environment. J. Infect. Dis..

[22] Teo T.-H., Ayuni N.N., Yin M., Liew J.H., Chen J.Q., Kurepina N., Rajarethinam R., Kreiswirth B.N., Chen L., Bifani P. (2024). Differential mucosal tropism and dissemination of classical and hypervirulent Klebsiella pneumoniae infection. Iscience.

[23] Liao C., Huang Y., Chang C., Hsu H., Hsueh P. (2014). Capsular serotypes and multilocus sequence types of bacteremic Klebsiella pneumoniae isolates associated with different types of infections. Eur. J. Clin. Microbiol. Infect. Dis..

[24] Catalán-Nájera J.C., Garza-Ramos U., Barrios-Camacho H. (2017). Hypervirulence and hypermucoviscosity: Two different but complementary Klebsiella spp. phenotypes?. Virulence.

[25] Hwang J.-H., Lee S.Y., Lee J., Hwang J.-H. (2022). Pyogenic spondylitis caused by Klebsiella pneumoniae: Should the possibility of hypervirulent Klebsiella pneumoniae be considered?. BMC Infect. Dis..

[26] Hwang J.-H., Handigund M., Hwang J.-H., Cho Y.G., Lee J. (2020). Clinical features and risk factors associated with 30-day mortality in patients with pneumonia caused by hypervirulent Klebsiella pneumoniae (hvKP). Ann. Lab. Med..

